# Kinematic Analysis of the Underwater Undulatory Swimming Cycle: A Systematic and Synthetic Review

**DOI:** 10.3390/ijerph191912196

**Published:** 2022-09-26

**Authors:** Santiago Veiga, Jorge Lorenzo, Alfonso Trinidad, Robin Pla, Andrea Fallas-Campos, Alfonso de la Rubia

**Affiliations:** 1 Grupo de Análisis Biomecánico, Departamento de Deportes, Facultad de Ciencias de la Actividad Física y del Deporte, Universidad Politécnica de Madrid, 28040 Madrid, Spain; 2 Deporte y Entrenamiento Research Group, Departamento de Deportes, Facultad de Ciencias de la Actividad Física y del Deporte, Universidad Politécnica de Madrid, 28040 Madrid, Spain; 3 Aqualab Research Group, Universidad Europea de Madrid, 28670 Madrid, Spain; 4 French Swimming Federation, 92110 Clichy, France; 5 Institut de Recherche bioMédicale et d’Epidémiologie du Sport (IRMES), 75013 Paris, France; 6 Núcleo de Estudios para el Alto Rendimiento y la Salud (ACUAUNA-NARS), Escuela Ciencias del Movimiento Humano, Universidad Nacional, Heredia 86-3000, Costa Rica

**Keywords:** performance, underwater filming, swimming start, swimming turn, segmental kinematics, angular kinematics, dolphin kick, competition

## Abstract

The increase of low-cost technology for underwater filming has made quantitative analysis an affordable resource for swimming coaches on a frequent basis. In this context, a synthesis of the kinematic determinants of underwater undulatory swimming (UUS) seems to be lacking. The aim of the present study was to synthesise the scientific evidence on the kinematic characteristics of competitive swimmers during UUS and the main kinematic determinants of UUS performance, as well as to summarise the main methodological considerations for UUS kinematic analysis. A systematic literature search was performed through four electronic databases following the PRISMA guidelines and STROBE for evaluating the quality of the included studies. Twenty-three research studies from the first search and two from the second search were finally considered. In total, 412 competitive swimmers (321 males and 91 females) with a performance standard of international B (11%), national (51%), or regional (35%) level were analysed. Most studies focused on a two-dimensional analysis of the ventral UUS performed from a push start and filmed 6–12 m from the starting wall. Kinematic analysis of UUS included kicking parameters (kicking length, frequency, and amplitude) as well as selected segmental kinematics in 76% of studies and the analysis of UUS performance determinants in 36%. Information about the determinants of UUS performance was inconsistent due in part to inconsistencies in the definition of kinematic parameters. Further research studies where automatic motion capture systems are applied to the analysis of UUS on the aforementioned conditions should be conducted.

## 1. Introduction

The underwater sections of swimming races are one of the most important components for competitive performance in that discipline. In these parts, swimmers achieve higher velocities than during surface swimming due to lower drag resistance [1] and the fast initial velocities achieved when diving (start) or pushing off the wall (turn). For this reason, the Federation Internationale de Natation (FINA) rules limit the duration of the underwater sections to the 15 m mark from the starting or turning wall. 

Distances travelled by elite swimmers in the underwater race sections typically range from 8 m to 14 m, depending on the event [2,3]. Recent research has reported that changes in the duration of underwater sections can have an important impact on the overall swimming performance [4]. The reason is the time gains due to faster forward velocity in these sections, but also the positive impact on the first swimming strokes after surface swimming resumption [5]. Indeed, swimmers of a higher level of skill displayed longer and/or faster underwater sections during competitive races than less proficient swimmers [6]. However, the extension of the underwater section can also lead to higher fatigue due to the hypoxic stimulus due to apnoea [7]; therefore, an appropriated strategy between velocity and energetics is essential.

The technique usually employed by swimmers during underwater sections is the underwater undulatory swimming (UUS), where athletes perform body undulations while keeping their arms outstretched and held together over the head [8]. The body wave movement travels caudally from the torso to the toes, increasing in amplitude at each body segment in a whip-like action [9]. Previous research has examined the propulsive mechanisms during human UUS, and it was revealed that the swimmers gained a thrust by creating vortices around the foot during the downward kick [10]. These vortices collide to form a strong water flow that provides propulsion [11]. 

The kinematic characteristics of competitive swimmers during UUS have been extensively reported, and, to a lesser degree, the determinants of better UUS performance have been examined. Faster vertical velocity of the toes during kicking [12], a greater range of motion (ROM) of the lower trunk during body undulations [13], and higher levels of symmetry in the downkick and upkick movements [9] have been related to faster forward velocities during UUS. In addition, kicking parameters, including kicking frequency, kicking length, and kicking amplitude, have been systematically measured in research studies. However, evidence from studies testing UUS is still inconsistent and somehow contradictory, as there is no clear consensus on what technical aspects should be prioritised when executing UUS for maximal velocity. For example, a simple kicking parameter such as kicking frequency has been correlated [14] and not correlated [13] to UUS velocity. 

The information about the kinematic execution of UUS seems to be relevant because coaches and practitioners can now employ affordable technologies for the evaluation of USS in their swimmers. The high-speed cameras integrated into smartphones or the waterproof devices represent a low-cost and user-friendly technology to perform qualitative or quantitative video analysis on a daily basis [15]. Therefore, a clear synthesis of the typical kicking parameters of competitive swimmers as well as the determinants of better UUS performance seems to be lacking for the swimming community, especially when referring to video-image methods that can be easily put into practice by coaches. This includes the synthesis of some methodological requirements that must be considered when analysing kinematics of UUS. 

While literature reviews have been conducted on the UUS topic, they have focused on the hydrodynamic aspects of UUS [16] or the determinant factors of UUS from a generic biomechanical, physiological, or neuromuscular perspective [17]. Therefore, the aims of the present study were (i) to synthesise the scientific evidence on the kinematic characteristics of competitive swimmers during UUS and the main kinematic determinants of human UUS performance and (ii) to summarise the main methodological considerations for UUS kinematic analysis. 

## 2. Materials and Methods

The stages of the procedure employed in completing the present systematic review adhered to both the Preferred Reporting Items for Systematic Reviews and Meta-Analysis (PRISMA) [18,19] checklist and the PROSPERO guidelines (registration no. 42021293041).

### 2.1. Search Strategy

Published studies connected to the kinematic analysis of the UUS cycle were identified through a systematic search of four electronic databases (MEDLINE via PubMed, Scopus, Sport Discus, and Web of Science) and by reviewing email alerts from research databases, with no restriction on publication date. The predefined search strategy was carried out using terms grouped into three search strings: (1) ‘professional swimmer*’ or ‘competitive swimmer*’ or ‘elite swimmer*’ or ‘high-level swimmer*’ or ‘international level swimmer*’ or ‘national level swimmer*’ or ‘amateur swimmer*’ or ‘semi-professional swimmer*’ or ‘university swimmer*’ or ‘collegiate swimmer*’ or ‘regional level swimmer*’ or ‘youth swimmer*’ or ‘junior swimmer*’ or ‘senior swimmer*’ or ‘master swimmer*’ or ‘skilled swimmer*’; (2) ‘underwater undulatory swimming’ or ‘human underwater undulatory swimming’ or ‘undulatory swimming’ or ‘underwater swimming’ or ‘underwater displacement’ or ‘underwater phase’ or ‘underwater dolphin kick’ or ‘submerged dolphin kick’ or ‘swimming gliding’ or ‘wave*’; (3) ‘kinematic*’ or ‘performance’ or ‘technique*’ or ‘coordination’ or ‘coordinative parameters’ or ‘determinant*’ or ‘velocity’ or ‘speed’ or ‘flexibility’ or ‘angle*’ or ‘angle* of attack’ or ‘swimming frequency’ or ‘kick frequency’ or ‘kick amplitude’ or ‘kick symmetry’ or ‘kick asymmetry’ or ‘trunk kinematic*’ or ‘chest angle*’ or ‘drag* parameter*’ or ‘joint range of movement’ or ‘range of motion’ or ‘cycle length’ or ‘cycle velocity’ or ‘propelling efficiency’ or ‘muscle strength’ or ‘rotation’ or ‘body alignment’.

### 2.2. Study Inclusion and Exclusion Criteria

Original scientific research based on the kinematic analysis of the UUS cycle in humans was considered. Studies were published in English or Spanish until April 2022 in peer-reviewed journals with an impact factor included in the Journal Citation Reports of the Web of Science (JCR of WoS).

According to the Population, Interventions, Comparisons, Outcomes, and Study Design (PICOS) question model, the inclusion criteria were as follows: (1) Population. All swimmers who participate in any standardised competition tier or level [20,21,22] were included: 1st level (‘A’ qualifying standards set to participate at international events, equivalent to ≥875 FINA points), 2nd level (‘B’ qualifying standards set to participate at international events, equivalent to 800–874 FINA points), 3rd level (qualifying standards set to participate at the national events, equivalent to 650–799 FINA points), 4th level (qualifying standards set to participate at the regional events, equivalent to 450–649 FINA points), and 5th level (participation in recreational events or <450 FINA points); (2) Intervention. Observational and/or experimental training and/or competition sessions with underwater filming or analysis; (3) Comparison. Recording of kinematic UUS parameters in swimmers belonging to control and/or experimental groups (inter-group and intra-group comparison); (4) Outcomes. Kinematic variables associated with the UUS cycle; (5) Study Design. Retrospective descriptive-observational study.

The exclusion criteria were (1) not including any kinematic parameters about the UUS (i.e., hydrodynamics); (2) focusing solely on parameters not defined by a kicking cycle (i.e., average velocities or times on pre-defined distances); (3) not including humans (i.e., computational or mathematical fluid analysis); (4) performing underwater swimming with techniques different than undulatory swimming (i.e., breaststroke pull-out or flutter kick); (5) performing underwater swimming using monofin or fins; (6) examining other swimming phases of the push or dive start (i.e., wall push-off, glide, surface swimming) exclusively; (7) including physically and/or intellectually disabled swimmers in the sample. Systematic reviews and other types of articles (i.e., conferences) associated with the analysis of kinematics parameters in UUS were only considered as support material in the search process for potentially valid research.

### 2.3. Data Extraction Strategy

The authors worked separately and independently to ensure the reliability of the process and the eligibility of the studies. According to the criteria for preparing systematic reviews (PRISMA) [18], the protocol was carried out in the months of November and December 2021. An update of the systematic search process was conducted on 30 April 2022 using the same methods and steps as in the first search. 

A standardised form was used to extract data from the studies included in the review for assessment of study quality and scientific evidence. Thus, the following information was collected: (A) author(s), year of publication, aim(s) of the study; (B) sample characteristics (n, gender and age); (C) sport context (competition level, records, and FINA points); (D) methodology and procedures applied (type of start, number of cameras, frame rate, swimmer position, distance to the wall, number of kick cycles, and experimental technique); and (E) main kinematic results on kicking parameters of USS (kicking velocity, kick length, kick rate, kick amplitude, and segmental kinematics) and kinematic determinants of UUS performance. In relation to the type of start, push or dive start referred to the participants beginning trials in the water and pushing off the wall or out of water from the starting block, respectively.

### 2.4. Study Quality Assesment

The Strengthening the Reporting of Observational Studies in Epidemiology (STROBE) checklist was used to determine the quality of the studies [23]. This checklist was composed of 22 items clustered into six categories belonging to the different study sections: Title–Abstract (item 1), Introduction (items 2 and 3), Methods (items 4–12), Results (items 13–17), Discussion (items 18–21), and Funding (item 22). A score of ‘0’ was assigned to incomplete items or items with a lack of information, and ‘1’ to items that were described accurately. The overall rating obtained from the sum of the item values was categorised according to the following levels: very low quality (0–4 points); low quality (5–8 points); medium quality (9–12 points); high quality (13–16 points); and very high quality (17–22 points). The study quality assessment was carried out by two independent reviewers (SV and JL). A third author (AR) resolved disagreements in the rating, and inter-rater reliability was calculated.

## 3. Results

### 3.1. Review Statistics

**Figure 1:**
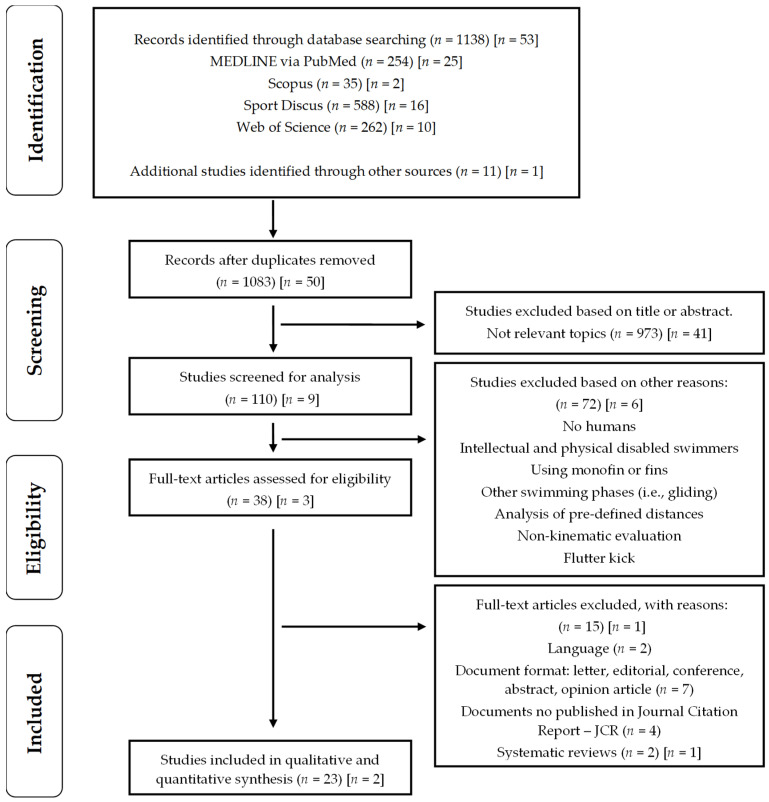
Flow diagram for screening and selection of studies according to Preferred Reporting Item for Systematic Reviews and Analysis (PRISMA). Notes: The brackets refer to the first search process (until December 2021) and the square brackets to the second process (from January to April 2022).

The four stages in both search processes, the first (1st) and second (2nd), can be seen separately in Figure 1. (1) Identification: the sixth author (AR) found 1138 (1st) and 53 (2nd) scientific studies in the four digital databases and 11 (1st) and 1 (2nd) additional studies identified through other sources (i.e., mail alert), totalling 1203 articles; (2) Screening: the sixth author (AR) eliminated the duplicate files in the first (*n* = 66) and the second search (*n* = 4), and the first author (SV) excluded those considered not relevant through a previous reading of the title, abstract, and keywords (1st search, *n* = 973; 2nd search, *n* = 41). Furthermore, the first author (SV), jointly with the second (JL), third (AT), and fourth authors (RP), rejected the studies not linked to the topic according to the exclusion criteria through a full-text reading (1st search, *n* = 72; 2nd search, *n* = 6); (3) Eligibility: the first (SV) and sixth authors (AR) eliminated full-text studies from the selection process by the eligibility criteria (1st search, *n* = 15; 2nd search, *n* = 1); (4) Inclusion: the remaining studies (*n* = 25), 23 from the first search and 2 from the second search, based on kinematic analysis of UUS were finally considered.

### 3.2. Study Characteristics

Table 1 shows the sample characteristics of the research studies, in chronological order, included in the present systematic review. In those studies where UUS results of male and female swimmers were presented separately, sample description was done by gender. In total, 412 swimmers (333 males and 79 females) were analysed during UUS, with 15 out of 25 studies providing the average FINA points of the swimmers’ personal best times. According to the standardised performance levels in swimming by Ruiz-Navarro et al. [22], none of the study samples belonged to level 1 (>875 FINA points), and only 35 swimmers (9.0%) in four research studies [12,24,25,26] were above 800 points, which is considered level 2. The greatest proportion of the study samples corresponded to level 3 (between 650 and 800 FINA points), with 89 swimmers from six studies, and to level 4 (450 to 650 FINA points), with 128 swimmers from five studies. The rest of the studies not specifying FINA points described their sample as national level in eight cases and regional level in one case [8]. In relation to the gender and age distribution of the sample, 13 out of 25 studies analysed male swimmers only, whereas one study [27] analysed female swimmers only. Swimmers in 8 out of 25 studies averaged under 18 years old, including one study [8] with swimmers between 10.6 and 11.6 years of age.

Table 2 synthesises the experimental procedures employed by research studies included in the present systematic review, with all studies focusing on the ventral UUS techniques from a push start, except two studies [28,29] examining UUS after a dive start. Swimmers were usually recorded from a lateral view to reconstruct two dimensions of their sagittal plane, considering the movement of the right and left sides of the body to be symmetrical. Experimental techniques employed for data processing were the scaling techniques and the direct linear transformation (DLT) algorithms [30], each employed in eight studies. Only two studies [26,31] calculated three-dimensional (3D) kinematics of UUS, with one performed in a water flume [26]. Additionally, two studies [8,32] did not employ video-image procedures but a linear encoder (speedometer).

Swimmers were instructed to perform maximum efforts of UUS ranging from 7.5 m [33] to 25 m [34,35,36,37], while video filming typically captured from 6 to 12 m away from the starting wall. Those kicking cycles included in the field of view of one camera (usually from one to three cycles) were included in the subsequent analysis, although Yamakawa et al. [26], who performed testing in a water flume, measured ten consecutive UUS kicking cycles. The analysis of UUS was focused on key (discrete) swimmer positions in nine studies, whereas 16 studies performed a continuous analysis of UUS. The filming frame rate was above 100 Hz in 15 out of 25 studies, and information about the shutter speed was provided by only four studies [13,16,36,38], with values ranging from 1/120 s to 1/1000 s. Magnitude of errors was reported by different calculations, such as standard error, maximal error, mean error, or root mean squared error, and it most typically ranged from 0.02 m to 0.04 m. In relation to the reliability of measurements (usually employed to estimate the manual digitalisation error), only three studies [9,12,29] reported explicit values of the error when repeatedly digitising one video sequence, with magnitudes falling within the aforementioned error range. For the automatic motion capture systems [26,31,39,40], the reported standard deviation in the dynamic calibration of a known length ranged between 1 and 2 mm.

### 3.3. Main Evidence on UUS Cycle Kinematics—Kicking Parameters

**Figure 2:**
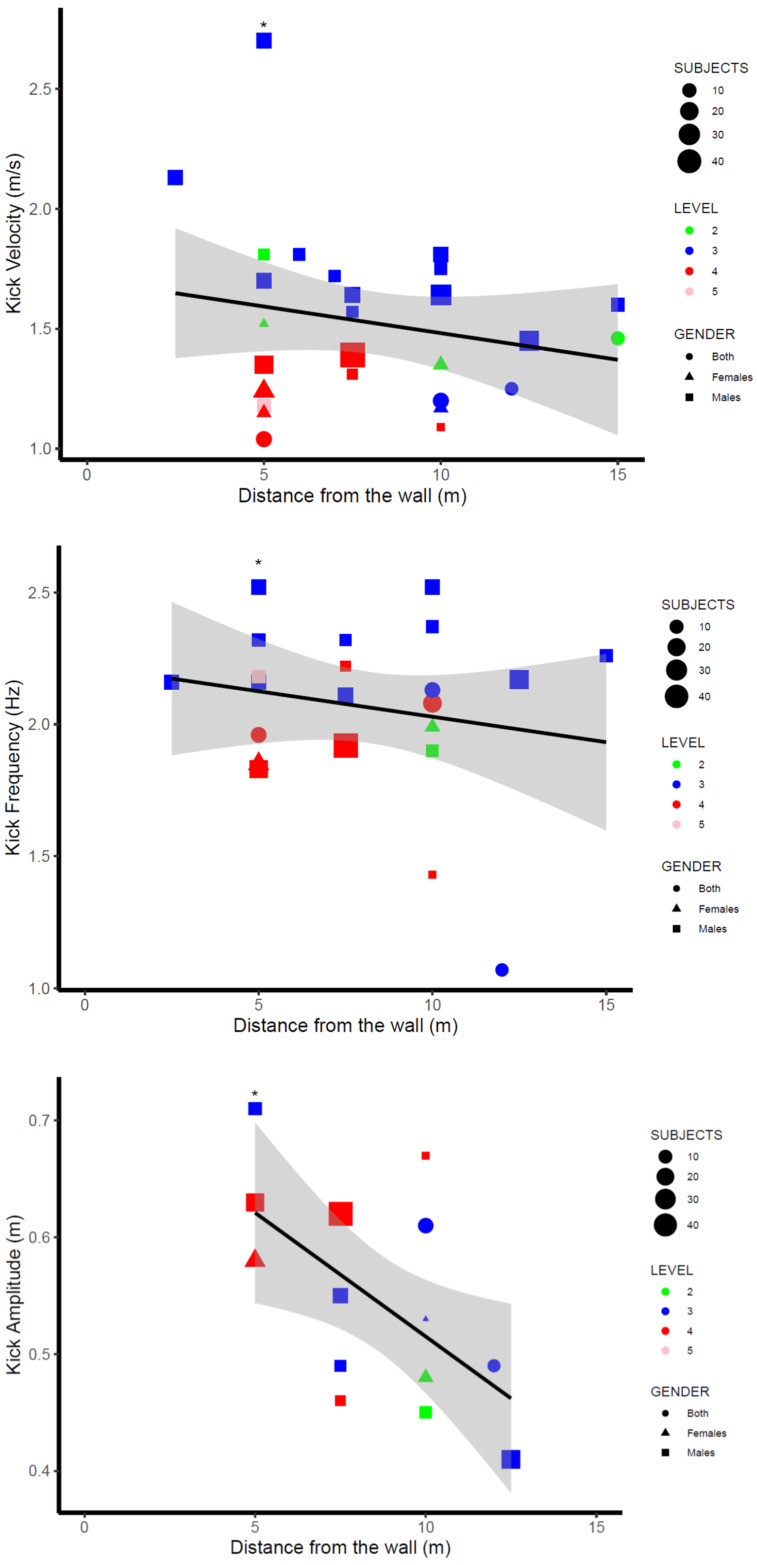
Evolution of kicking parameters during UUS according to distance from the starting wall. Note: data from a dive start are denoted with *.

**Figure 3:**
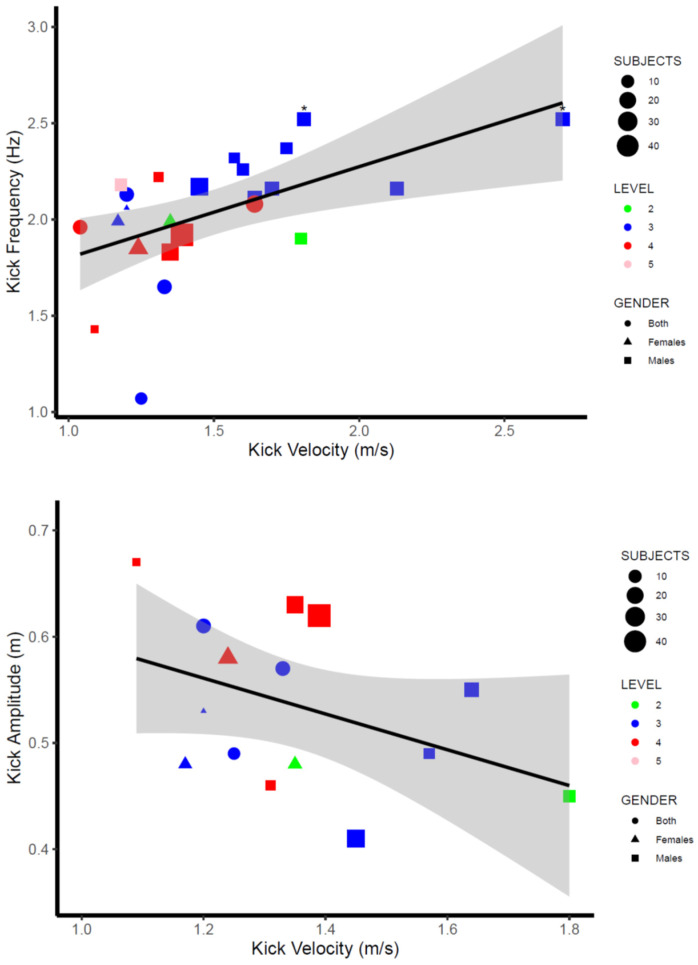
UUS kicking parameters in relation to the kicking velocity. Note: data from a dive start are denoted with *.

**Figure 4:**
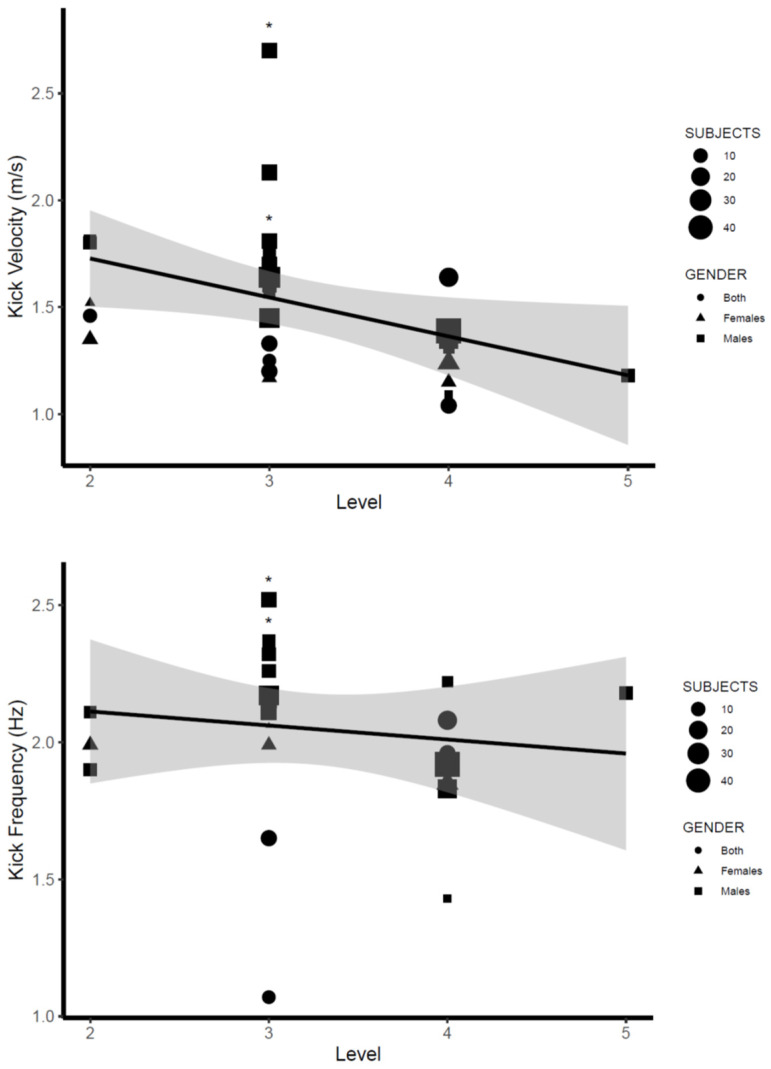

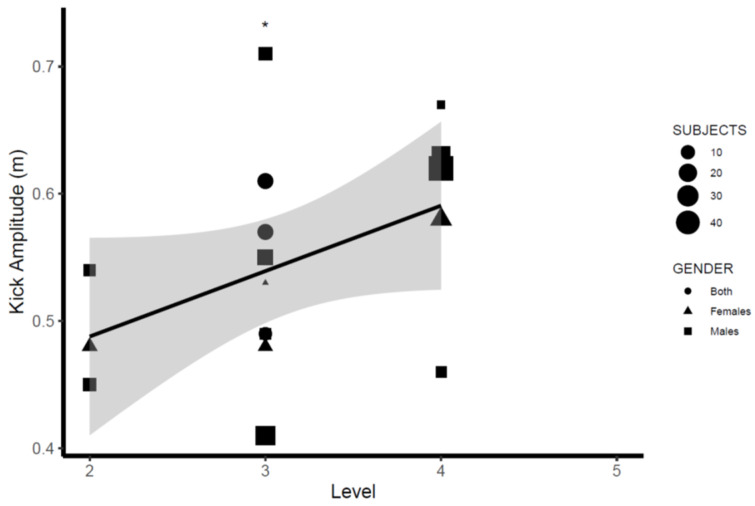
UUS parameters in relation to the swimmers’ level of skill. Note: data from a dive start are denoted with *.

Table 3 compiles the main kicking parameters (kicking velocity, frequency, length, and amplitude) obtained in the research studies included in the present systematic review. Values reported for kicking velocity ranged from 1.09 m/s [27] to 2.70 m/s [29] in male swimmers and from 1.15 m/s [32] to 1.52 m/s [12] in female swimmers. Kicking length values ranged from 0.57 m [16] to 0.82 m [41] in swimmers from both genders, whereas kicking frequencies ranged from 1.43 Hz [27] to 2.52 Hz [29] in males and from 1.83 Hz [42] and 2.06 Hz [43] in females. Finally, kicking amplitude measured in reference to the toes ranged from 0.41 m [31] to 0.70 m [28] in males and from 0.48 m [25,27] to 0.58 m [42] in females. A comprehensive synthesis of kicking parameters in relation to the level of skill, gender, and distance from the starting wall is illustrated in Figure 2, Figure 3 and Figure 4 (see Section 4).

**Table 1:** Characteristics of the sample (author, year, gender, age, competitive level) and aims of the studies related to the kinematic analysis of UUS cycle.

Author	Year	Males	Females	Age	Competitive Level	FINA Points	Aim(s) of the Study
Collard et al. [34]	2011	6	5	18.0 ± 3.0	Level 3—National	-	To compare the UUS performance between the anguilliform-like and carangiform-like techniques.
Hochstein & Blickhan [43]	2011	-	2	25.0	Level 3—National	-	To analyse the kinematics of swimming athletes during UUS.
Zamparo et al. [35]	2012	7	5	20.5 ± 5.2	Level 2—International B	-	To analyse the efficiency of the dolphin kick in determining the velocity and acceleration in the first 5 m and the following 10 m after a turn (v5, v5–15, a5, and a5–15) in a 100 m simulated front crawl race.
Houel et al. [28]	2013	10	-	21.4 ± 4.5	Level 3—National	-	To determine the kinematics variables that improve performance during the underwater phase of grab starts.
Atkison et al. [9]	2014	15	-	21.5 ± 3.2	Level 3—National	663.0 ± 134.0	To determine how sagittal kick symmetry in the underwater dolphin kick between the downkick and upkick phases is related to underwater dolphin kick performance.
Hochstein & Blickhan [27]	2014	1	7	21.6	Level 3—National	-	To find out to what extent the human swimmer approaches an ideal undulatory wave which is symmetric with respect to the extended gliding position.
Shimojo et al. [44]	2014	10	-	21.3 ± 0.9	Level 3—National	-	To investigate whether changing the kick frequency while maintaining UUS at maximal effort would change the other UUS kinematics, such as swimming velocity and propelling efficiency, in well-trained male swimmers.
Willems et al. [41]	2014	15	11	16.4 ± 2.5	Level 4—Regional	595.0 ± 121.0	To investigate the effect of ankle flexibility and muscle strength on dolphin kick performance in competitive swimmers.
Connaboy et al. [16]	2016	8	-	17.6 ± 1.4	Level 3—National	-	To determine which kinematic variables were key to the production of maximal UUS velocity.
		-	9	16.4 ± 0.8			
Higgs et al. [12]	2017	7	3	21.1 ± 2.6	Level 2—International B	812.0 ± 66.0	To determine which kinematic variables of the upbeat and downbeat are associated with prone UUS performance in an elite sample.
Yamakawa et al. [25]	2017	-	8	20.9 ± 1.9	Level 2—International B	817.6 ± 18.2	To investigate the effects of increased kick frequency on the propelling efficiency during underwater dolphin kick.
Shimojo et al. [40]	2019	8	-	19.7 ± 1.1	Level 3—National	713.1 ± 42.1	To investigate the Froude (propelling) efficiency and three-dimensional (3D) kinematics of human UUS following the extrinsic restriction of the ankle by tape application.
		-	9	19.6 ± 0.8			
Wadrzyk et al. [42]	2019	23	-	16.8 ± 0.6	Level 4—Regional	533.0 ± 66.0	To determine gender-related differences of UUS kinematic indicators and their impact on UUS velocity.
		-	18	16.7 ± 0.6		551.0 ± 68.0	
Gonjo & Olstad [29]	2020	14	-	19.8 ± 2.5	Level 3—National	686.0 ± 85.7	To establish relationships between selected underwater kinematics and the starting and turning performances and to quantify kinematic differences between these segments in sprint butterfly swimming.
Matsuura et al. [24]	2020	9	-	20 ± 2	Level 2—International B	821.1 ± 68.2	To identify muscular coordination in the trunk and lower limb during UUS in elite swimmers.
Takeda et al. [38]	2020	8	-	19.6 ± 1.2	Level 3—National	733.6 ± 57.5	To investigate the deceleration effect of flutter kicking after dolphin kicking before commencing the stroke at swimmer’s emersion.
Crespo et al. [32]	2021	10	-	16.6 ± 2.0	Level 5—Recreational	402.0 ± 120.0	To assess the effects of an activation protocol based on post-activation performance enhancements upon UUS; and evaluate the differences between males and females.
		-	7	15.4 ± 1.8	Level 4—Regional	483.0 ± 102.0	
Ikeda et al. [13]	2021	9	-	20.4 ± 1.67	Level 3—National	766.0 ± 91.4	To identify the kinematic variables associated with dolphin kick performance during the acceleration and deceleration phases.
Matsuda et al. [31]	2021	26	-	22.0 ± 2.7	Level 3—National	714.1 ± 103.7	To investigate the relationship between 3D lower-limb kinematics and forward-swimming velocity during UUS at maximal velocity.
Ruiz-Navarro et al. [8]	2021	10	-	11.6 ± 0.2	Level 4—Regional	-	To evaluate the effects of a training protocol on UUS and underwater gliding performance and kinematics in young swimmers.
		-	7	10.6 ± 0.4			
Stosic et al. [36]	2021	30	-	16.8 ± 1.4	Level 3—National	-	To examine the role of segmental, kinematic, and coordinative parameters on the swimming velocity during the pre-transition and transition phases.
Wadrzyk et al. [33]	2021	47	-	17.2 ± 1.01	Level 4—Regional	553.0 ± 94.0	To establish relationships between somatic build and kinematic indices describing UUS.
Stosic et al. [37]	2022	33	-	16.5 ± 1.3	Level 3—National	-	To examine the effect of the breakout movements on the stroking variables and coordinative patterns of competitive swimmers.
Tanaka et al. [39]	2022	7	-	20.6 ± 2.40	Level 4—Regional	626.2 ± 81.2	To compare the foot and trunk kinematic parameters during UUS between faster and slower swimmers.
Yamakawa et al. [26]	2022	8	-	21.1 ± 1.0	Level 2—International B	800.4 ± 81.4	To investigate the changes in kinematics and muscle activity with increasing swimming velocity during UUS.

Note: Sample data are split in gender groups when available. Competitive level according to classification by Ruiz-Navarro et al. [22].

**Table 2:** Experimental procedures of the studies on the kinematic analysis of the UUS cycle.

Author	Year	Body Position	Start Type	Wall Distance (m)	Kick Cycles (n)	Frame Rate (Hz)	Cameras (n)	Experimental Technique—Software
Collard et al. [34]	2011	Ventral	Push Start	12	1	60	1	Two-dimensional linear scaling—Dartfish ProSuite
Hochstein & Blickhan [43]	2011	Ventral	Push Start	10	1	30	1	Automatic marker tracking—WinAnalyse V1.0
Zamparo et al. [35]	2012	Ventral	Push Start	15	3	25	1	Two-dimensional linear scaling—SIMI motion
Houel et al. [28]	2013	Ventral	Dive Start	Entire underwater (0–10 m)		25	3	Modified double plane direct linear transformation—SIMI motion
Atkison et al. [9]	2014	Ventral	Push Start	7.5	3–5	30	1	Two-dimensional linear scaling—Human Movement Analysis software
Hochstein & Blickhan [27]	2014	Ventral	Push Start	10	2	30–125	2	Two-dimensional automatic marker tracking—WinAnalyse 2.1.1
Shimojo et al. [44]	2014	Ventral	Push Start	15	3	100	2	Two-dimensional direct linear transformation—Tracker
Willems et al. [41]	2014	Ventral	Push Start	10	3	300	3	Angle tool—Kinovea 0.8.15
Connaboy et al. [16]	2016	Ventral	Push Start	10	6	50	1	Two-dimensional linear scaling—APAS-2000
Higgs et al. [12]	2017	Ventral	Push Start	5	3–6	100	1	Two-dimensional linear scaling—Wetplate
Yamakawa et al. [25]	2017	Ventral	Push Start	10	3	100	2	Two-dimensional direct linear transformation—Tracker
Shimojo et al. [40]	2019	Ventral	Push Start	0	3	60–120	6	Two-dimensional direct linear transformation—FRAME-DIAS 4Three-dimensional automatic motion capture—VENUS-3D
Wadrzyk et al. [42]	2019	Ventral	Push Start	5	3	120	1	Linear Scaling-Skill Spector
Gonjo & Olstad [29]	2020	Ventral	Dive Start	Entire underwater section		50	10	Two-dimensional automatic motion analysis—AIM
Matsuura et al. [24]	2020	Ventral	Push Start	10	3	200	2	Two-dimensional direct linear transformation—Tracker
Takeda et al. [38]	2020	Ventral	Push Start	6	3	59.96	1	Two-dimensional direct linear transformation (DLT)—Tracker
Crespo et al. [32]	2021	Ventral	Push Start	5	4	200	0	Speedometer Heidenhain
Ikeda et al. [13]	2021	Ventral	Push Start	10	2	120	1	Two-dimensional linear scaling—FrameDIAS V
Matsuda et al. [31]	2021	Ventral	Push Start	12.5	3	200	17	Three-dimensional automatic motion capture—Oqus Underwater
Ruiz-Navarro et al. [8]	2021	Ventral	Push Start	5	3–6	200	0	Speedometer
Stosic et al. [36]	2021	Ventral	Push Start	10	1	50	2	Two-dimensional direct linear transformation
Wadrzyk et al. [33]	2021	Ventral	Push Start	7.5	3	120	1	Linear Scaling—Skill Spector
Stosic et al. [37]	2022	Ventral	Push Start	10	1	50	2	Two-dimensional direct linear transformation
Tanaka et al. [39]	2022	Ventral	Push Start	7.5	3	100	8	Three-dimensional automatic motion capture—Qualysis
Yamakawa et al. [26]	2022	Ventral	Water flume		4	100	18	Three-dimensional automatic motion capture—VENUS-3D

**Table 3:** Kicking parameters of the UUS cycle.

Author	Year	Gender	Kicking Parameters				Segmental Kinematics	UUS Performance Determinants
			Kicking Velocity (m/s)	Kick Length (m)	Kick Rate (Hz)	Kick Amplitude (m)		
Collard et al. [34]	2011	Both	1.25 ± 0.29	0.53	1.07 ± 0.19	0.49 ± 0.08	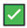	
Hochstein & Blickhan [43]	2011	Females	1.20 ± 0.06		2.06 ± 0.10	0.53 ± 0.03	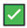	
Zamparo et al. [35]	2012	Both	1.46 ± 0.15	0.71 ± 0.12			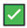	
Houel et al. [28]	2013	Males			2.32 ± 0.22	0.70 ± 0.04		
Atkison et al. [9]	2014	Males	1.64 ± 0.15	0.79 ± 0.08	2.11 ± 0.18	0.55 ± 0.07	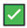	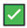
Hochstein & Blickhan [27]	2014	Males	1.09 ± 0.11		1.43 ± 0.54	0.67 ± 0.20		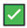
		Females	1.17 ± 0.04		1.99 ± 0.27	0.48 ± 0.06	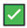	
Shimojo et al. [44]	2014	Males	1.60 ± 0.12	0.71 ± 0.06	2.26 ± 0.16		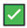	
Willems et al. [41]	2014	Both	1.64 ± 0.20	0.82 ± 0.21	2.08 ± 0.40		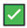	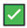
Connaboy et al. [16]	2016	Both	1.20 ± 0.13	0.57 ± 0.07	2.13 ± 0.23	0.61 ± 0.07	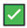	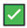
Higgs et al. [12]	2017	Males	1.81 ± 0.32		2.27 ± 0.45		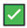	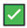
		Females	1.52 ± 0.23					
Yamakawa et al. [25]	2017	Females	1.35 ± 0.08		1.99 ± 0.15	0.48 ± 0.05	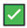	
Shimojo et al. [40]	2019	Both	1.33 ± 0.19		1.65 ± 0.18	0.57 ± 0.06	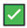	
Wadrzyk et al. [42]	2019	Males	1.35 ± 0.15		1.85 ± 0.26	0.63 ± 0.07	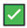	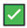
		Females	1.24 ± 0.12		1.83 ± 0.20	0.58 ± 0.06		
Gonjo & Olstad [29]	2020	Males	2.70 ± 0.27		2.52 ± 0.23		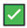	
			1.81 ± 0.15					
			2.13 ± 0.21		2.16 ± 0.19			
			1.70 ± 0.11					
Matsuura et al. [24]	2020	Males	1.80 ± 0.20		1.90 ± 0.30	0.45 ± 0.06	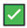	
Takeda et al. [38]	2020	Males	1.77 ± 0.12					
		Males	1.76 ± 0.13					
Crespo et al. [32]	2021	Males	1.18 ± 0.08		2.18 ± 0.33			
		Females	1.15 ± 0.11					
Ikeda et al. [13]	2021	Males	1.75 ± 0.16		2.37 ± 0.23		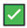	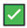
Matsuda et al. [31]	2021	Males	1.45 ± 0.15	0.68 ± 0.09	2.17 ± 0.33	0.41 ± 0.06	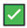	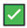
Ruiz-Navarro et al. [8]	2021	Both	1.04 ± 0.16		1.96 ± 0.24			
Stosic et al. [36]	2021	Males		0.77 ± 0.12	2.14 ± 0.35	0.31 ± 0.06	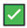	
Wadrzyk et al. [33]	2021	Males	1.39 ± 0.18	0.73 ± 0.09	1.92 ± 0.28	0.62 ± 0.08		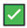
Stosic et al. [37]	2022	Males	1.62 ± 0.17					
Tanaka et al. [39]	2022	Males	1.57 ± 0.15	0.69 ± 0.08	2.32 ± 0.40	0.49 ± 0.05	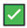	
			1.31 ± 0.09	0.58 ± 0.07	2.22 ± 0.29	0.46 ± 0.07		
Yamakawa et al. [26]	2022	Males	1.43 ± 0.10	0.68 ± 0.08	2.11 ± 0.33	0.54 ± 0.05	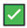	

Note: average values for different gender, skill level or distance to the wall groups are indicated in separate rows within the same study.

### 3.4. Main Evidence on UUS Cycle Kinematics—Segmental Kinematics

The information about UUS was complemented in 19 out of 25 studies (76%) with selected segmental kinematics of the kicking motion. The position of the joint centres and the angular motion of the feet, leg and thigh, trunk, and arm segments [45] were typically reported on the swimmers’ key positions. However, some studies performed a more in-depth analysis of trunk motion with additional points in the body mechanical model. For example, Atkinson et al. [9] and Tanaka et al. [39] considered the chest, upper waist, and lower waist segments (divided by xiphoid, lower end of tenth rib, and iliac horn, respectively), whereas other studies [13,24,25,44] analysed the kinematics of the lower end of the rib to divide upper and lower trunk. Finally, studies specifically examining the flexibility of the ankle joint considered additional points in the foot complex such as the seven points monitored in Willems et al. [41].

Ikeda et al. [13] reported the position of body landmarks in relation to the swimmer’s hip centre, and it was indicated that the highest and lowest body parts during the UUS cycle were the ankle (23 cm above hip) and knee (11 cm below hip) at the instant of minimum velocity, and the shoulder (2 cm above hip) and knee (21 cm below knee) at maximum velocity. The vertical displacement of joint centres during kicking motion indicated a progressive increase in amplitude from the proximal to the distal joints. Specifically, vertical amplitude of the head–shoulders was between 0.07 m and 0.11 m [16,27,34], hip 0.13 m, knee 0.26 m to 0.27 m, and ankle 0.42 m to 0.46 m [16,27]. The time lag between the maximum positions of joint centres allowed to calculate the so-called body wave velocity, with values between 2.68 m/s [40] and 4.61 m/s [12], which represented the speed of the caudal momentum transfer during body undulation.

The swimmers’ body inclination was represented by the trunk inclination (defined by shoulder and hip line) and adopted increasing values from the first kick (3° to 7° in turn or start, respectively) to the last kick before emersion, where values of 12° [29] and 16° [36] were reported. The angular position of key joints involved in the UUS movement such as shoulder or hip did not deviate greater than 30° from a straight line, both in the instant of maximal and minimal velocity [13,24]. Knee flexion at upkick obtained values lower than 60°, whereas maximal hyperextension at downkick achieved −11° in national-level swimmers [24,41]. Range of movement of joints between the UUS key positions achieved 28° in the shoulder joint [16], between 30° and 50° in the hip joint [16,26,31], between 71° and 89° in the knee joint, and between 34° and 64° in the ankle joint [16,26,31,42]. For the trunk segments, ROM for the lower trunk, upper trunk, and chest was characterised as 27°, 12°, and 19°, respectively, in national-level swimmers [39]. 

Velocity of the kicking motion was also described in several studies [12,13,16,26,39,40] with instantaneous kinematics of UUS that allowed to calculate peak angular velocity of selected joints in both the upkick and downkick motions. Maximal angular velocities were 624°/s [13] and 702°/s [16] in the knee joint, whereas minimum angular velocities were around 122°/s [13] or 100°/s [39] for the upper trunk segment. Kicking velocity was also described by the vertical velocity of the toes during both downkick and upkick, with values in males between −3.61 m/s [12] and −4.07 m/s [26] in the downkick and between 3.16 m/s [25,39] and 4.10 m/s [12] in the upkick. On the other hand, values reported for the toe vertical velocity in females were −3.26 m/s (downkick) and 2.74 m/s (upkick) [25].

### 3.5. Main Evidence on UUS Cycle Kinematics—Determinants of Kicking Performance

Nine studies (36%) sought relationships between the kicking kinematic parameters and UUS performance. In chronological order, Atkinson et al. [9] demonstrated a high relationship between kicking symmetry in the sagittal plane and UUS performance. A better performance on the upward kicking was related to greater knee hyperextension, lower knee flexion at upkicking, lower ‘dorsal flexion’ at downkick, and, consequently, faster angular velocity of the toes when kicking upwards. In the same line, Hochstein and Blickan [27] observed that faster swimmers at UUS were able to diminish asymmetry on the joint movements along the body longitudinal axis. Connaboy et al. [16] observed that maximal angular velocity of the knee and ankle and ROM of the knee explained a great proportion of UUS performance (R^2^ = 0.939). However, a great part of variance of UUS velocity depended on the participants’ fixed effect, highlighting the influence of individual technique on UUS performance. Higgs et al. [12] detected that maximal vertical toe velocity in conjunction with wave body velocity could predict UUS velocity (R^2^ = 0.775). Wadrzyk et al. [42] observed that maximal ankle extension, kicking length, and horizontal feet displacement during kicking were related to forward velocity during kicking, although some differences were observed between genders. All the mentioned studies performed two-dimensional analysis of the kinematic characteristics of UUS. 

Recently, Matsuda et al. [31] described for the first time the 3D kinematic characteristics of UUS and reported that the maximum angular velocity of hip rotation during kicking was related to the horizontal velocity of swimmers. At the same time, a greater active range of movement of hip rotation was related to greater hip rotation velocity during kicking. Ikeda et al. [13], in a 2D study of UUS, observed that the angular displacement of the lower trunk was correlated with a greater angular motion of the shoulder, knee, and lower leg segments during kicking and, consequently, with a greater horizontal velocity of swimmers during UUS. In addition, the ankle, end of rib, and shoulder vertical positions relative to the hip were related to the UUS velocity. Finally, Willems et al. [41] demonstrated a relationship between strength levels in the ankle joint and the UUS velocity, specifically the strength on the plantar flexion and the internal rotation of the ankle.

### 3.6. Study Quality

The quality analysis (RAE–Performance Strengthening the Reporting of Observational Studies in Epidemiology (STROBE) checklist) yielded the following results: (a) The quality scores ranged from 10 to 18. (b) The average score was 15 points. (c) A total of 16 out of the 25 included studies (64%) were categorised as high quality (13–16 points), and 7 (28%) were considered very high quality (17–20 points). The highest scores were located in the Abstract (100%), Introduction (98.0%), Discussion (94.0%), Method (75.4%), and Other Information (68%) sections. The lowest scores were detected in the Results section (56.0%). Among the highest-quality studies, items 1 (abstract/summary), 2 (background/rationale), 5 (participants), 8 (data source—procedure for determining performance measurement), 16 (key results concerning study objectives), 18 (overall interpretation of results considering objectives and relevant evidence), and 19 (generalisability of the study results) were considered complete (100%). By contrast, the most uncommon items were numbers 12 (statistical estimate and precision for each sample or subgroup group examined are provided) with 56.0%, 14 (a measure of effect size) with 40.0%, 13 (main results—post hoc comparisons [OR] between grouping category) with 24.0%, and 10 (handle of duplicates or missing data) with 4.0%.

## 4. Discussion

The present systematic review aimed to synthesise the kinematic characteristics of competitive swimmers during UUS and the main kinematic determinants of human UUS performance according to research studies in journals with impact factor (JCR of WoS). In addition, the experimental procedures employed for the kinematic analysis of the UUS cycle were compiled. Results indicated that UUS performance has not been explored in world-class or international-level swimmers [21] and that a third of publications analysing national-level swimmers did not indicate the sample FINA points. From the five standardised performance levels suggested by Ruiz-Navarro et al. [22], 217 swimmers (more than half of the total sample) belonged to levels 3 and 4. According to the classification by Mckay et al. [21], most of the sample analysed in the present systematic review would place in tiers 2 and 3, named as trained–developmental or highly trained–national levels and far from elite-level tiers 4 and 5. In addition, caution should be taken when interpreting results due to different standards in performance classification across countries [22].

### 4.1. Study Characteristics

Typical data collection made swimmers push off the pool wall in the streamline position to achieve a depth between 0.5 and 1 m [9,45], unless performing race [29] or dive [28] simulations. Interestingly, most studies instructed swimmers to maintain themselves in the same water depth for the entire underwater section and to avoid using the push start to maximise forward velocity. In this way, researchers tried to standardise experimental conditions and to avoid a positive influence of the push-off velocity (close to 3 m/s if performed maximally [35,46,47]) on the subsequent underwater kicking. After pushing off, most studies constrained swimmers to perform maximal UUS efforts on 15 m distances, although trials ranged from 7.5 m [42], 10 m [8,28,32,41], 12 m [33], 20 m [12,31,45], to 25 m [34,35,36,37]. Only Takeda et al. [38] and Stosic et al. [36,37], in testing conditions, and Gonjo and Olstad [29], in a race simulation, made swimmers maintain the maximal swimming efforts after emersion from under the water. The rationale for most studies was to isolate the underwater kicking movements to accurately evaluate the kicking skill of participants apart from other start or turn subsections. However, it should be considered that UUS does not happen in isolation during competitive races, and, therefore, a proper transition from underwater to surface swimming seems to be paramount for the overall performance [37]. 

UUS movements were recorded in most studies (11 out of 25) between 6 and 12 m off the starting wall. This represents an important methodological decision, as kinematic changes have been reported during underwater segments, probably due to fatigue [48]. For example, Houel et al. [28] described a decrement in forward velocity during UUS of approximately −0.5 m/s for each 1.5 m of the underwater section, and Gonjo and Olstad [29] reported a 25% velocity decrease from the first to the last underwater kick of the start and turn of a simulated 50 m butterfly race. Additionally, Taladriz et al. [48] and de Jesus et al. [49] observed a 15% decrease in kicking velocity from the beginning to the end of the underwater swimming sections. Changes in the kicking velocity in relation to the starting distance could be coupled with changes in the kicking kinematics; therefore, this could affect the evaluation of the kicking parameters, body posture, or angular kinematics during UUS. The qualitative interpretation of Figure 2, where kicking parameters are displayed according to the distance from the starting wall, suggested a decrease in kicking amplitude from the 5 m to the 12 m mark, as well as a tendency to decrease in kicking frequency and kicking velocity from 2.5 m to 15 m. However, no further evidence in the literature has been reported according to the publications included in the present systematic review. 

The number of cameras employed for filming depended on each research aim, but 11 out of 25 studies employed only one camera for underwater recordings, and its positioning was located between 8 m [33,41] and 12 m [16] away from the swimmers’ sagittal plane. Connaboy et al. [45] stated that three cycles should be needed to obtain reliable values of the kicking kinematics, and this recommendation was followed by ten studies, although two kicking cycles [13,27], or even one [34,36,37,43], were also monitored in seven studies. This methodological decision somehow contradicts the fact that competitive swimmers usually perform between 8 and 12 kicks in the underwater segments of start and turns [6], and it assumes that the selected kicking cycles are representative of the entire underwater section. An alternative approach was employed by Shimojo et al. [44], who filmed the UUS with two cameras in sequence and described a correction in the kinematic data to avoid the shift in the camera optical axis. In relation to the temporal resolution (or frame rate) of filming, all research studies until 2014 reported recording frame rates between 25 Hz and 60 Hz. However, from then on, 13 out of 19 research studies employed at least 100 Hz, with 100–120 Hz being the most common selection. This is in line with frame rates reported in research about swimming starts [50] or turns [51].

Some studies implemented automatic motion capture systems in recent years to avoid the manual marking of the swimmers’ position on the screen, for both race analysis [29] and laboratory conditions [26,31,39,40]. This allowed a time-saving continuous analysis of the kicking motion trajectories, but a higher number of cameras to capture swimming motions that ranged from 5 above and 5 under water [29] to 18 under water [26]. Filtering procedures for the continuous raw data of UUS typically consisted of a low-pass Butterworth filter with cut-off frequencies that ranged from 4 to 7 Hz, with 6 Hz being the most common frequency. These cut-off frequencies are usually employed in swimming motion [52], although they are lower than those employed for the swimming starts [50]. The calculation of real space coordinates from the screen (digitised) coordinates was performed by either mean of linear scaling techniques or DLT algorithms [30] in 16 out of 25 studies. DLT algorithms allowed correcting for perspective errors between the camera axis and the swimmers’ plane of movement, which made this procedure recommended in those experimental set-ups where the swimmers’ position was not centred in the camera’s field of view.

The information about the validity of kinematic analysis was indicated in most of the research studies included in the present systematic review. However, up to ten studies did not explicitly indicate the accuracy or reliability of their measurements, which represents a clear methodological limitation. In relation to calibration error, several studies reconstructed known lengths within the field of view of underwater recordings, including the swimmers’ thigh employed for scaling in four studies [9,12,13,35]. The uncertainty of the spatial measurements during UUS seemed to fall within 0.04 m, which represents ≈5% of a typical kicking length for competitive swimmers. This level of uncertainty was greater than previously reported for 3D underwater analysis of swimming motions [33], although it seemed to be decreased when automatic motion capture systems were employed [26,31,39,40].

### 4.2. Kicking Parameters

Kicking velocity values of competitive swimmers were faster than typical surface swimming velocities, except in those cases where the last kicks of underwater sections were measured. Indeed, both Takeda et al. [38] and Stosic et al. [37] reported that forward velocity on the last underwater kick before emersion was slower than the first arm-stroke cycle of swimming breakout [53]. This was probably related to the decrease in forward velocity experienced by swimmers during the underwater section [28], and it highlighted the importance of adjusting the duration of the UUS sections before emersion to surface swimming. Previous research in World Swimming Championships suggested the greater importance of average velocity rather than the distance travelled underwater by elite swimmers in 100 m event races [4], which could reinforce the notion of avoiding overly long underwater sections. 

The dynamics of kicking velocity with the kicking parameters are illustrated in Figure 3, and it suggests a linear increase of kicking frequency with kicking velocity at the same time as a decrease of kicking amplitude with velocity. These data are in line with Yamakawa et al. [26], who compared the UUS characteristics of national-level swimmers at their 70%, 80%, and 90% maximal velocities and observed an increase in the kicking frequency at the same time as a decrease in the kicking length and amplitude. In addition, the vertical toe velocity and its upkick–downkick symmetry as well as the angular joint velocity were reported to increase with UUS velocity. However, a comprehensive understanding of the dynamics of kicking parameters would probably require calculating values relative to the swimmers’ size, as previously done by Shimojo et al. [44] and Hochstein and Blickhan [27]. According to them, swimmers should aim to increase kicking frequency without decreasing relative kicking amplitude to maximise UUS velocity, as kicking velocity relative to body length could be more heavily related to the kicking frequency (r = 0.86) than to the kicking amplitude (r = −0.45) [54]. The importance of the kicking parameters related to body size would also rely on data indicating that both fish [55] and competitive swimmers [43] presented similar kicking amplitude relative to body length (between 0.2 and 0.3 body lengths) at preferred kicking frequencies.

Based on the rationale that kicking frequency could serve as a control parameter over kicking velocity and also that applied forces during UUS could increase linearly with kicking frequency [56], some studies examined the effect of deliberately increasing the frequency of kicking motions above the spontaneous values. However, supramaximal frequencies between 105% and 115% did not positively affect kicking velocity but decreased the kicking propelling efficiency [44] and the efficiency of the muscular co-activation patterns involved in kicking motion [25]. Relationships of the kicking frequency with forward velocity in UUS were generally small to medium (between 0.2 and 0.67 [28,42]), which suggests that other segmental variables related to the kicking motion and the propelling efficiency, as well as the swimmers’ anthropometrics, could be involved in the achievement of maximal kicking velocity. 

### 4.3. Segmental Kinematics

The position of body landmarks during UUS was usually characterised during two key positions that divided the kicking motion into two phases, upkick and downkick, corresponding to the movement between the highest and lowest vertical coordinates of the swimmers’ toe during body undulation movements [9] or also defined as the time between the beginning and the end of the downward movement (or upward) of the foot segment [31]. Arellano et al. [14] considered two additional subphases within the upward kicking according to the predominantly vertical or horizontal movement of the feet. This was done due to the relationship between the transition point of the upkick and the horizontal velocity of the swimmer’s centre of mass. Nevertheless, no studies in the present literature review, except two [26,45], further considered these two upkick subphases for analysis. Kicking phases depending on the acceleration or deceleration of the swimmer’s centre of mass were also considered by Ikeda et al. [13], although this approach seemed to be in line with the downkick and upkick phases. Finally, based on previous work by Colman et al. [57] and in order to investigate the role of ankle flexibility, Willems et al. [41] considered additional key positions in the kicking motion such as the instant of maximal plantar flexion of the ankle. This position occurred slightly later than the highest position of the toes at upkick (as swimmers were already pushing down water with kicking), and it represented large differences in the ankle joint angular position. Therefore, researchers should take into account the slightly different definitions in the upkick positions that could explain large inter-study differences observed in the ankle joint range of movement of UUS.

Data collected on the segmental kinematics of competitive swimmers confirmed the UUS as a body wave movement that travelled caudally in a whip-like action [9]. Additionally, (1) the vertical displacement of joint centres during kicking motion from the head (0.07–0.11 m) to the ankle (0.42–0.46 m) [16,27,34], (2) the range of movement of the key joints (from 28° in the shoulder to 71°–89° in the knee) [16,31,39,42], and (3) the maximal angular velocities of the kicking motion (around 700°/s in the knee joint versus values close to 100°/s for the upper trunk segment) [13,16,39] increased their magnitude from the torso to the toes. An important role on the body undulation seemed to be played by the lower trunk segment, defined by the lower end of the tenth rib and the iliac horn [13]. A greater range of movement of this segment would favour a greater movement of the lower leg for kicking propulsion, despite not modifying the knee range of movement [13,39]. In addition, a lower position of the end of the rib in relation to the hip would be related to faster forward velocities [13]. Body undulation during USS was also characterised as body wave velocity, calculated from the time lag between the minimum or maximum position of key anatomical landmarks and the distance between them [40]. More specifically, the whole-body wave velocity was defined as the slope of regression for the shift in the minimum position along the body axis depending on time [25,43,45]. This parameter represented a quantification of the speed of the caudal momentum transfer during body undulation, and it had been previously related to the forward swimming velocity in both marine animals [58] and competitive swimmers [54], as it would indicate the efficiency of the undulatory movement for propulsion [35]. According to the studies included in the present systematic review, the ratio between body wave velocity and forward UUS velocity would fall between 2.0 and 2.8, suggesting the importance of fast body undulation movements. 

### 4.4. Determinants of Kicking Performance

**Figure 5:**
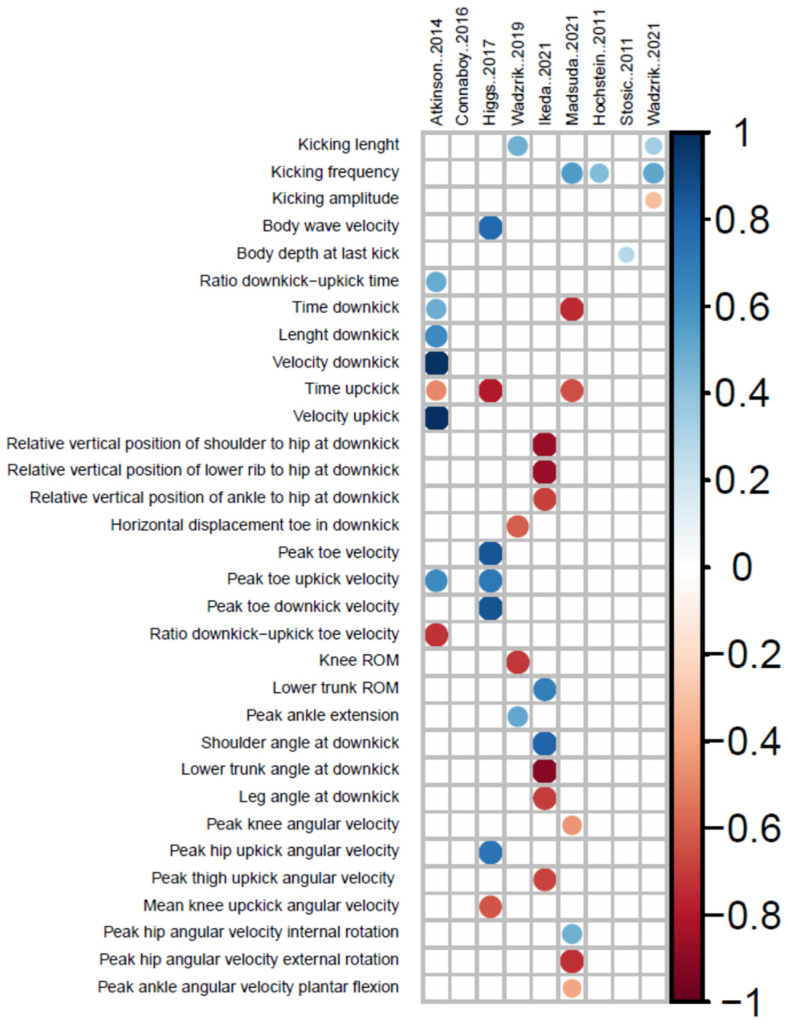
Correlation matrix between kinematic parameters and UUS performance [9,12,13,16,31,33,36,41,43]. Note: Blue colour indicate positive correlation coefficient, whereas red indicate increasing negative correlation coefficient. Color intensity and the size of the circle are proportional to the correlation coefficients.

In relation to the determinants of UUS velocity, as illustrated in Figure 5, the lack of coherence in the relationships observed between the kinematic parameters and the UUS performance made drawing solid conclusions difficult. Firstly, there was no clear consensus on what kicking parameter (frequency, length, or amplitude) showed the strongest relationship with the UUS velocity. Secondly, there was a lack of studies examining the role of segmental kinematics on the centre of mass kinematics during UUS, with Ikeda et al. [13] performing the most comprehensive analysis. Finally, the definition of angular parameters considerably differed between studies, probably related to the experimental procedures employed. Some studies differentiated the upkick and downkick parameters, while others focused on specific body segments and/or joints, and there was no consensus on whether average or peak segmental velocities should be calculated. Of course, two-dimensional kicking parameters such as frequency or amplitude are easier to collect and to explain to swimmers, but other parameters in both two and three dimensions could provide valuable information for UUS performance and should be systematically collected in UUS research studies.

As observed in Figure 5, only the kicking length [33,42] and kicking frequency [31,33,43], the duration of the upkick and downkick phases [9,12,31], and the peak toe upkick velocity [9,12] were significantly related to UUS performance in more than one study. This represents that, from 32 kinematic parameters with statistical relationships to UUS performance, only five of them were reported in more than one study. Despite the lack of agreement, some insights could be deduced from data: the peak toe velocity during upkick was related to the forward acceleration of the swimmers’ center of mass as it could be linked to the timing and magnitude of vortex generation for propulsion [9,12]. The peak of velocity should occur early during the upkick and it would be facilitated by the hip extension (rather than knee) movement. Faster movement of the end effector during upkick would probably assist swimmers in decreasing the duration of upkick in relation to downkick, which was related to faster UUS [9,12]. In relation to kicking parameters, relationships between longer kicking length and better UUS performances were only detected in groups of low competitive level [33,42]. On the other hand, the influence of attaining higher kicking frequency on the UUS velocity seemed to rely on swimmers maintaining the kicking efficiency [25,45] and relative amplitude [27,45], which would explain small to medium correlations of kicking frequency displayed in Figure 5. Further research including up-to-date experimental procedures where standardised kinematic parameters could be consistently related to UUS performance would be welcome. Wadzryk et al. [33] reported that the influence of swimmers’ somatic structure on UUS performance was small and that the kicking technique was the main factor influencing UUS effectiveness. Therefore, solid relationships between the kinematic characteristics of UUS and performance should still be unveiled by researchers.

### 4.5. Differences by Groups of Gender, Level of Skill, Body Position, and Type of Start

Although some evidence on the kinematic determinants of UUS performance was revealed, there are still several gaps in the knowledge about UUS execution. First of all, there are no studies examining the kinematic determinants of UUS in the dorsal body position, although the backstroke swimmers present the longest underwater distances during the start and turns of elite competitions [4]. Back in 1999, Arellano et al. [59] showed no statistical differences in kicking velocity or any of the kicking parameters between the ventral and dorsal positions. Only small differences were detected in shoulder, trunk, or knee angular positions at the upkick and downkick positions. Later, in 2006, Alves et al. [60] observed greater vertical oscillations and also a greater ankle extension in the dorsal compared to the prone position. However, there is no evidence in the literature about the performance determinants of dorsal undulatory kicking and whether these determinants differ from the ventral position. 

Secondly, there is little information on the gender-related differences in UUS execution. Wadrzyk et al. [42] compared the kinematic characteristics of youth males and females during short maximal efforts, but the competitive level of the sample was around 550 FINA points, which would be classified as level 4 in a 1–5 scale [22]. In that study, males displayed 9% faster UUS velocity than females, with a greater kicking amplitude and kicking length, but no differences in kicking frequency were observed. Interestingly, females showed greater ankle ROM than males. Therefore, caution should be taken when pooling data on both genders until more evidence of the specific gender differences in UUS are explored. Gender differences in the swimming performance have been explained by differences in the strength levels [61] and anthropometrics [62], with males presenting up to 33% greater strength levels and a greater height, arm span, and hand size that could favour their better performance. On the other hand, female swimmers have been characterised by a lower drag force and a more economical swim at submaximal intensities [63]. Considering these specific gender traits, specific female UUS analysis would be welcome. 

In relation to differences in the UUS execution according to the level of skill of swimmers, most evidence was reported in conference articles [14,64,65] and suggested differences in the body undulation movement between more and less skilled swimmers. Hochstein and Blickhan [27] reported that more skilled swimmers presented a lower amplitude in the body undulation, especially in the arm/hand region, and a more symmetric movement of joints along the body axis. Connaboy et al. [64] observed greater levels of variability in the phase angles during body undulation for less skilled swimmers. Wang et al. [65] and Tanaka et al. [39] reported greater velocities in the kicking motion for skilled swimmers with a greater peak of angular velocity on the chest, lower trunk, thigh, and shank segments and also a faster vertical velocity of the toes during both upkick and downkick. In addition, a greater ROM of the lower trunk was also observed. However, differences in the body undulation seemed not to have a clear effect on the kicking parameters. Arellano et al. [14] detected differences in kicking velocity and kicking frequency (up to 20% difference) between international- and national-level swimmers, but no changes in the kicking amplitude, kicking length, or kicking vertical velocity. On the other hand, Tanaka et al. [39] observed that more skilled swimmers displayed almost 30 cm longer kicking length but no greater kicking frequency or amplitude than less skilled swimmers. Overall, the qualitative interpretation of average kicking parameters in the research studies (Figure 4) indicates a clear decrease in kicking velocity for swimmers of a lower level of skill coupled with a trend for lower kicking frequency and greater kicking amplitude for swimmers classified as tiers 4 and 5 (regional and recreational levels). These data are in line with previous findings about kicking amplitude [27] and suggest a better control of the body undulation for faster swimmers.

Finally, the type of start could also affect the kinematic characteristics of the kicking motion. As observed in Figure 2, UUS kicking parameters after a dive start (denoted with *) were notably greater than after push start. Gonjo and Olstad [29] reported 20% faster velocity in the first underwater kicking after the starting dive than the wall push-off. This could be explained by velocity differences when diving off the starting block [50] compared to pushing off the wall [46]. In addition, the swimmer’s body position was closer to horizontal when kicking after pushing off the wall, and this could be coupled with other segmental differences. Nevertheless, no further evidence on the UUS after dive start was detected, as 23 out of 25 research studies in the present systematic review were performed with push start. 

### 4.6. Future Research on USS

Further research should be conducted on UUS where international-level swimmers are analysed in the entire underwater segments with automatic motion capture systems. This could include a comparison with swimmers of a different level of skill, which would inform on how segmental kinematics evolve during kicking cycles. Considering the relatively simple motion patterns performed during UUS, automatic motion capture systems would provide important time savings during data processing and also a lower error rate. The proposed experimental set-up should be conducted to measure standardised segmental parameters of the kicking motion, which would allow solid conclusions about the kinematic determinants of UUS. In addition, studies focusing on UUS in the dorsal position and in the specific gender differences in the kicking motion would be welcome to narrow the knowledge gap about UUS execution in competitive swimmers. Once some light could be shed on these questions, further aspects of UUS, such as the longitudinal changes from youth to senior swimmers or the benefits provided by racing suits, should also be examined.

## 5. Conclusions

The present systematic review highlighted the lack of scientific evidence on some essential aspects of UUS, such as the kinematic characteristics of UUS in the dorsal position or the specific differences in UUS execution between genders and swimmers of different levels of skill. Kicking parameters provided by the reviewed studies indicated an inverted relationship between kicking frequency and amplitude in relation to velocity, with a linear increase of the kicking frequency and a decrease of kicking amplitude with kicking velocity. Additionally, swimmers of a higher skill presented faster velocities and higher kicking frequencies but a lower kicking amplitude. Information about the kinematic determinants of UUS was inconsistent in part due to inconsistencies in the kinematic parameters measured during UUS execution. In addition, there was a lack of evidence on changes in UUS execution according to the distance to the wall and the type of start, although the synthesis of the different sample groups suggested a decrease in kicking amplitude, frequency, and velocity from 5 to 15 m. Further research studies where automatic motion capture systems (with a lower measurement error rate) are applied to the analysis of world-class swimmers in both ventral and dorsal UUS during the entire underwater sections should be conducted. 

## Data Availability

The raw data supporting the conclusions of this article will be made available by the authors, without undue reservation.
